# Trapeziectomy Versus Carpometacarpal Arthroplasty for Basilar Thumb Arthritis: A Narrative Review of Comparative Outcomes

**DOI:** 10.7759/cureus.96871

**Published:** 2025-11-14

**Authors:** Kavyansh Bhan, Dhruvi Chande, Nirav Valand, Janani Suresh, Shradha Prakash, Satyanarayana Pidikiti

**Affiliations:** 1 Trauma and Orthopedics, University Hospitals Dorset NHS Foundation Trust, Bournemouth, GBR; 2 Orthopedics, Hinduhridaysamrat Balasaheb Thackeray (HBT) Trauma Centre, Mumbai, IND; 3 Trauma and Orthopedics, Barking, Havering and Redbridge University Hospitals NHS Trust, Romford, GBR

**Keywords:** thumb basal joint arthroplasties, thumb carpometacarpal osteoarthritis, thumb cmc arthroplasty, trapeziectomy, trapezium-metacarpal joint

## Abstract

Basilar thumb arthritis or thumb carpometacarpal (CMC) joint osteoarthritis is a common degenerative condition causing pain, weakness, and functional limitation, particularly among older populations and postmenopausal women. Surgical intervention is often required for advanced disease, with trapeziectomy and prosthetic arthroplasty being the two main approaches. Trapeziectomy, with or without ligament reconstruction and tendon interposition (LRTI), provides reliable long-term pain relief and functional restoration. Prosthetic arthroplasty aims to preserve thumb length, maintain biomechanics, and enable faster rehabilitation. This review synthesizes evidence comparing trapeziectomy-based procedures and CMC arthroplasty, focusing on pain, functional outcomes, strength, range of motion, complications, implant survival, rehabilitation, and patient satisfaction. Both approaches achieve excellent outcomes, with arthroplasty offering earlier functional gains but higher implant-specific complications and revision rates. Trapeziectomy remains a durable, cost-effective, and predictable treatment option. Individualized patient selection and shared decision-making are emphasized, and long-term comparative studies are needed to refine surgical guidelines.

## Introduction and background

Thumb carpometacarpal (CMC) joint osteoarthritis is a prevalent source of hand disability, particularly affecting women in the fifth to seventh decades of life [1,2]. It can cause progressive pain at the base of the thumb, reduced grip and pinch strength, and limitations in daily activities such as writing, opening jars, or key manipulation. The trapeziometacarpal joint is a saddle-shaped articulation that allows complex multiplanar motion, including flexion, extension, abduction, adduction, and circumduction. Degenerative changes often include cartilage loss, osteophyte formation, subluxation, and joint instability, resulting in pain and functional impairment [1-4].

Initial management is conservative, including splinting, analgesics, nonsteroidal anti-inflammatory drugs (NSAIDs), corticosteroid injections, and hand therapy to maintain range of motion and strengthen intrinsic and extrinsic musculature [4,5]. When conservative measures fail, surgical intervention is indicated to relieve pain, restore function, and preserve thumb biomechanics [5,6].

Trapeziectomy, with or without ligament reconstruction and tendon interposition, is the historical gold standard, offering reproducible pain relief and functional improvement [7-9]. Its main limitations include thumb shortening, loss of leverage, and a relatively longer rehabilitation period. Prosthetic arthroplasty has emerged as an alternative, aiming to preserve joint height and kinematics, potentially allowing earlier return to function [10,11]. Implant designs range from silicone spacers to modern metal-polyethylene, pyrocarbon, and dual-mobility devices, each with unique advantages and potential complications [12-14]. This review evaluates the comparative evidence for trapeziectomy and CMC arthroplasty, focusing on functional outcomes, strength, pain relief, range of motion, complications, implant survival, rehabilitation, patient satisfaction, and cost considerations [15-17].

## Review

Methods

A narrative literature review was conducted using PubMed, Scopus, and Google Scholar databases to identify English-language studies published between October 2010 and October 2025. Search terms included “thumb carpometacarpal arthritis,” “trapeziectomy,” “ligament reconstruction tendon interposition,” “CMC arthroplasty,” and “prosthesis.” Systematic reviews, randomized controlled trials, and comparative cohort studies evaluating functional, pain, strength, cost-effectiveness, and complication outcomes were included. Reference lists of key articles were also reviewed to identify additional relevant studies.

Pain and functional outcomes

Achieving adequate pain relief remains one of the principal goals of any CMC arthritis surgery. Both trapeziectomy and arthroplasty achieve significant and durable reductions in visual analog scale (VAS) pain scores [1-3]. Trapeziectomy with or without ligament reconstruction and tendon interposition (LRTI) provides predictable long-term pain control, with multiple studies demonstrating maintained relief beyond five years [7-9,18]. Arthroplasty also reliably reduces pain, with some evidence suggesting more rapid early pain improvement due to maintenance of thumb length and joint stability [10,12,14].

Functional recovery is assessed using validated patient-reported outcome measures (PROMs), such as the Disabilities of the Arm, Shoulder, and Hand (DASH) questionnaire and the Michigan Hand Questionnaire (MHQ) [19,20]. Both trapeziectomy and arthroplasty show significant improvements in PROMs at 12 months and beyond [7-9,19]. While some studies indicate slightly better early function in arthroplasty, these differences typically resolve over time, resulting in comparable long-term functional outcomes [10,12,21]. High patient satisfaction rates are reported for both techniques, provided surgical indications and patient expectations are appropriately managed [9,21].

Strength and range of motion

Thumb strength, including key pinch and grip, is a crucial functional outcome. Trapeziectomy leads to moderate improvements in key pinch and progressive enhancement of grip strength [9,22]. Arthroplasty preserves lever mechanics, often demonstrating superior early key pinch and slightly enhanced early grip strength, although these differences generally disappear by 12 months [12,14,23]. Range of motion, particularly opposition and abduction, is slightly better in the early postoperative phases following arthroplasty; however, long-term motion outcomes are largely equivalent between procedures [14,24]. The choice of implant design and surgical technique can influence these outcomes, with suture suspension arthroplasty and dual-mobility implants showing favorable early ROM [11,24].

Complications and revision rates

Trapeziectomy and LRTI are associated with generally minor complications, including tendon donor-site morbidity, scar sensitivity, transient sensory changes, and rarely complex regional pain syndrome [5,9]. Arthroplasty-specific complications include dislocation, loosening, subsidence, implant wear, and periprosthetic fracture [10,12,14]. Although overall complication rates may be similar, arthroplasty complications are more likely to necessitate revision surgery [10,12,14]. Revision rates for trapeziectomy remain low, often below 5% [7-9], whereas arthroplasty revisions vary by implant type, ranging from 5% to 20% over medium-term follow-up [12,14,24]. Careful patient selection, meticulous surgical technique, and postoperative monitoring are critical for minimizing complications and subsequent revisions.

Implant survival and longevity

Trapeziectomy demonstrates durable outcomes with minimal need for revision and sustained pain relief and function [7-9]. Arthroplasty survival is highly implant-dependent; modern devices, such as ARPE, Moovis, and PyroDisk, demonstrate 80-95% survivorship at eight to 10 years [12,14,23]. Factors influencing implant survival include bone quality, surgical technique, patient activity level, and implant design [10,25]. Awareness of long-term risks is essential when counseling younger or high-demand patients considering arthroplasty who may be candidates for different surgical procedures, including CMC fusion.

Rehabilitation and return to function

Rehabilitation is integral to optimizing outcomes for both procedures. Following trapeziectomy, immobilization in a thumb spica splint or cast is typically maintained for four to six weeks, followed by structured hand therapy focusing on range of motion, strengthening, and gradual load progression [25,26]. Arthroplasty often allows earlier mobilization, with light activity resumption within six to eight weeks [4,12,21]. Tailored rehabilitation protocols enhance recovery of function and improve patient satisfaction, regardless of surgical technique [26].

Cost considerations

Trapeziectomy is generally more cost-effective due to low implant costs and minimal need for revision surgery [25]. Arthroplasty entails higher upfront costs related to implants and potential revision procedures, although earlier return to work may offset some economic considerations [27,28]. A cost-benefit analysis should consider patient-specific factors, such as age, occupation, and physical activity demands [28].

Table 1 provides a comprehensive comparison of trapeziectomy and carpometacarpal arthroplasty across multiple outcome domains, including pain relief, functional scores, strength, range of motion, complications, revision rates, time to functional recovery, and patient satisfaction. Both procedures achieve substantial long-term improvements in pain and function, with arthroplasty offering slightly faster early gains in key pinch strength and range of motion. Complication profiles differ, with trapeziectomy-associated issues generally minor, whereas arthroplasty carries implant-specific risks and higher revision rates. Overall, the table highlights that while early postoperative advantages may favor arthroplasty, long-term outcomes and patient satisfaction remain comparable, supporting individualized surgical decision-making.

**Table 1:** Comparative outcomes of trapeziectomy and carpometacarpal arthroplasty across key functional domains. VAS: visual analog scale; DASH/MHQ: Disabilities of the Arm, Shoulder, and Hand/Michigan Hand Questionnaire; ROM: range of motion; CRPS: complex regional pain syndrome

Outcome domain	Trapeziectomy	Arthroplasty	Key points
Pain relief	Marked reduction in VAS, durable [1-3,7]	Marked reduction, faster early relief [1,4,12]	Long-term pain relief equivalent; early benefit with arthroplasty [1,4,12]
Functional scores (DASH/MHQ)	Significant improvement, sustained [7-9,21]	Significant improvement; early gains possible [4,12,21]	Similar function at 12 months; early advantage for arthroplasty [4,12,21]
Key pinch strength	Moderate improvement [9,22]	Higher early improvement [12,14,23]	Short-term pinch advantage arthroplasty; equal long-term [12,14,23]
Grip strength	Progressive improvement [9,22]	Comparable improvement [9,22]	Long-term grip strength is similar [9,22]
Range of motion	Good restoration [7,24]	Slightly better early ROM [14,24]	Early ROM advantage arthroplasty; long-term equivalent [14,24]
Complications	Donor morbidity, scar, CRPS [5,9]	Implant-specific: dislocation, loosening [10,12,14]	Similar overall, arthroplasty may need revision [10,12,14]
Revision/survival	Low revision, durable [7-9]	Implant-dependent 80-95% at 8-10 years [12,14,23]	Trapeziectomy rarely revised; arthroplasty higher revision [12,14,23]
Time to functional return	10-12 weeks [25,26]	6-8 weeks [4,12,21]	Faster early return arthroplasty [4,12,21]
Patient satisfaction	High, durable [7-9]	High; dependent on implant survival [12,14,23]	Both high; long-term favors durable trapeziectomy [7,12]

Discussion

This review demonstrates that both trapeziectomy/LRTI and CMC arthroplasty reliably relieve pain and restore function. Early postoperative advantages of arthroplasty include improved pinch strength, slightly better range of motion, and earlier functional recovery, which may be particularly relevant for younger or high-demand patients [4,12,14,23]. Long-term outcomes, however, demonstrate comparable pain relief, functional gains, and patient satisfaction between the two procedures [7-9,21].

Trapeziectomy offers a predictable, durable, and arguably cost-effective option, particularly for older patients or those with lower functional demands [7-9,27]. Its minimal implant dependence translates to lower long-term risk of revision. Arthroplasty is attractive for patients seeking earlier return to activity and preservation of thumb mechanics, but implant-related complications must be carefully considered [10,12,14,23]. Patient selection should account for age, activity level, bone quality, and expectations. Surgical expertise and implant familiarity play critical roles in optimizing outcomes. Rehabilitation is essential for both procedures, with structured therapy facilitating recovery of strength, mobility, and function [25,26].

Limitations

The evidence base is heterogeneous, with variability in study design, follow-up duration, implant types, and rehabilitation protocols. Randomized head-to-head trials remain limited. Despite these factors, consistent findings support excellent cost-effective outcomes for both techniques [27,28]. Further long-term comparative studies are warranted to guide optimal procedure selection and refine implant designs [29,30].

## Conclusions

Trapeziectomy with or without LRTI and CMC arthroplasty both provide reliable pain relief, functional restoration, and high patient satisfaction. Arthroplasty offers earlier recovery and short-term strength advantages but carries a higher risk of implant-specific complications and revision. Trapeziectomy remains a durable, predictable, and cost-effective procedure. Individualized surgical decision-making, informed patient counseling, and structured rehabilitation are key to optimal outcomes.
